# Enhancing Inpatient Echocardiogram Request Appropriateness and Timeliness Using British Society of Echocardiography Guidelines: A Quality Improvement Project

**DOI:** 10.7759/cureus.116614

**Published:** 2026-09-20

**Authors:** Abdurrahman Yusuf, Benjamine Ajoku, Ritika Sharma, Davida Commey

**Affiliations:** 1 Cardiology, East Suffolk and North Essex NHS Foundation Trust, Colchester, GBR; 2 Internal Medicine, Colchester Hospital, Colchester, GBR; 3 Cardiology, Colchester Hospital, Colchester, GBR

**Keywords:** british society of echocardiography, clinical audit, inpatient care, quality improvement projects, transthoracic echocardiogram

## Abstract

Introduction

Inpatient echocardiography is an important component of cardiovascular assessment, but inappropriate requests and inadequate clinical information may complicate prioritisation and delay diagnostic imaging. This quality improvement project aimed to improve the appropriateness of inpatient echocardiography requests in accordance with British Society of Echocardiography (BSE) guidance and to improve the timeliness of echocardiogram completion.

Methodology

This was an uncontrolled before-and-after quality improvement study conducted at Colchester Hospital, England, using two measurement points. The baseline audit covered 15-24 March 2025, and the reaudit covered 6-15 June 2025. The latest 100 eligible inpatient echocardiography requests from each period were reviewed using a pragmatic sampling approach. Pre-operative and peri-operative requests and requests relating to patients who had been discharged before audit assessment were excluded. The intervention, delivered between April and May 2025, comprised clinician education, dissemination of BSE guidance and baseline findings by email, educational posters, and teaching sessions. Appropriateness was assessed against BSE recommendations by a single cardiology consultant blinded to the audit period. Time to completion was measured from the recorded request timestamp to echocardiographic image acquisition.

Results

Appropriate requests increased from 82/100 (82%) at baseline to 90/100 (90%) at reaudit, an absolute change of 8 percentage points (95% CI −1.7 to +17.8; Fisher's exact p=0.153). Completion within 24 hours changed from 77/100 (77%) to 79/100 (79%), an absolute change of 2 percentage points (95% CI −9.5 to +13.4; Fisher's exact p=0.865). The median time to completion was 14.8 hours (IQR 5.6-21.4) at baseline and 17.5 hours (IQR 3.3-23.4) at reaudit. Retrospective BSE urgency adjudication classified 71/100 (71%) baseline and 77/100 (77%) reaudit requests as urgent. Among urgent requests, 55/71 (77.5%) at baseline and 59/77 (76.6%) at reaudit were completed within 24 hours.

Conclusion

Following the implementation of a guideline-based educational intervention, an increase in request appropriateness was observed, while overall completion within 24 hours changed only modestly. The findings support continued use of guideline-based education and iterative audit while highlighting the need to evaluate service-level and process measures when assessing echocardiography timeliness.

## Introduction

Echocardiography is an important non-invasive cardiovascular imaging modality that provides information regarding cardiac structure and function and plays a central role in the assessment of a broad range of cardiovascular conditions. In the inpatient setting, timely imaging may influence diagnosis, treatment, and clinical decision-making.

Despite its clinical utility, inappropriate referrals and requests containing insufficient clinical information can complicate echocardiography triage. Diagnostic requests should communicate relevant clinical findings and provide a clear clinical question to the receiving service. Previous research has demonstrated deficiencies in the completeness, accuracy, and coherence of echocardiography referral forms, highlighting the potential importance of improving referral processes and clinician education [1].

The British Society of Echocardiography (BSE) provides guidance on clinical indications and prioritisation of inpatient echocardiography [2]. The guidance distinguishes between studies that are not indicated as inpatient investigations, indicated but non-urgent studies, urgent studies, and emergency studies. Urgent inpatient studies should generally be undertaken within 24 hours of referral, whereas emergency studies should generally be undertaken within 60 minutes. These recommendations provide a framework for prioritising requests according to anticipated clinical value and clinical time sensitivity.

Educational interventions have previously been associated with improvements in the appropriate use of echocardiography. Bhatia et al. demonstrated that an appropriate use criteria-based educational intervention reduced inappropriate outpatient echocardiography requests and increased appropriate requests [3]. Levitt et al. similarly reported improved appropriate use of stress echocardiography following an educational strategy and decision-support intervention [4]. A subsequent systematic review and meta-analysis found that quality improvement interventions, including education and decision support, were associated with reductions in rarely appropriate echocardiography [5].

At Colchester Hospital, England, concerns were identified regarding the quality and appropriateness of inpatient echocardiography requests, including requests that did not require inpatient investigation and requests lacking sufficient clinical information to support effective triage. A baseline audit was therefore undertaken against BSE recommendations.

The theory of change was that improving clinicians' knowledge of appropriate indications, documentation requirements, and urgency categories would improve the quality of submitted requests. Better request quality was expected to support more consistent prioritisation by cardiac physiologists (echocardiographers), potentially reducing avoidable delays associated with clarification or inappropriate requests. However, the project did not directly measure triage time, request-return rates, physiologist workload, or echocardiography capacity.

The quality improvement project aimed to increase the proportion of appropriate inpatient echocardiography requests from 82% to 90% and to increase the proportion of echocardiograms completed within 24 hours from 77% to 85%.

## Materials and methods

Study design and setting

This was an uncontrolled before-and-after quality improvement study with two measurement points conducted at Colchester Hospital, England. The project was registered as a clinical audit under audit reference MEDCOL24-034 and quality improvement reference QIP 25-639. The project comprised a baseline audit conducted from 15 to 24 March 2025, an intervention period from April to May 2025, and a reaudit conducted from 6 to 15 June 2025.

Study population and sampling

The study population comprised inpatient echocardiography requests submitted to the echocardiography service during the respective audit periods. A pragmatic sampling approach was used. The latest 100 eligible inpatient echocardiography requests available during each audit period were selected for analysis. No formal random sampling technique was used. During the baseline sampling process, 107 requests were screened to obtain 100 eligible requests; seven were excluded because the patients had been discharged and were therefore no longer inpatients at the time of audit assessment. During the reaudit sampling process, 105 requests were screened to obtain 100 eligible requests; five were excluded because the patients had been discharged and were therefore no longer inpatients at the time of audit assessment. Pre-operative and peri-operative echocardiography requests were excluded. Requests relating to patients who had already been discharged before audit assessment were also excluded.

Audit standards and definition of appropriateness

The audit standards were based on BSE guidance on clinical indications and triage of emergency, inpatient, and critical care echocardiography [2]. For this project, a request was considered appropriate when the documented clinical indication supported inpatient echocardiography and was consistent with relevant BSE recommendations. The assessment also considered whether the request contained sufficient clinical information and a clinically meaningful reason for investigation, whether the requested study was appropriate to the clinical context, and whether repeat or potentially unnecessary inpatient imaging was justified by the documented clinical circumstances.

Appropriateness was assessed using the information documented on the original request form. Information subsequently communicated verbally to the echocardiography service was not retrospectively added to the written request when determining request quality.

All requests were assessed by one cardiology consultant. The assessor was blinded to whether the request belonged to the baseline or reaudit period at the time of appropriateness assessment. No formal inter-rater assessment was performed because only one assessor was used; consequently, Cohen's kappa could not be calculated.

Retrospective BSE urgency classification

Requests were retrospectively classified according to four BSE categories: emergency, generally requiring echocardiography within 60 minutes; urgent, generally requiring echocardiography within 24 hours; indicated but non-urgent, appropriate for inpatient echocardiography without a defined 24-hour requirement; and not indicated, inpatient echocardiography not supported by the BSE guidance. This classification was a retrospective adjudication based solely on the clinical information documented on the request form. It was not the contemporaneous triage decision made by the echocardiography service.

Intervention

A PDSA-based intervention was implemented between April and May 2025 following review of the baseline audit findings. The intervention consisted of 20 informal educational teaching sessions, each lasting approximately 20-30 minutes. Teaching was delivered by the quality improvement lead and targeted medical staff involved in submitting inpatient echocardiography requests.

Teaching focused on appropriate indications, documentation of relevant clinical history and examination findings, urgency classification, and BSE recommendations. Education also covered appropriate investigation of suspected heart failure, including consideration of B-type natriuretic peptide (BNP) testing where clinically indicated.

Baseline audit findings and relevant BSE guidance were disseminated by email to medical staff. Educational posters and visual reminders were placed in clinical areas. Attendance was not formally recorded, and the precise number and locations of posters were not formally documented. No planned changes to echocardiography staffing, physiologist sessions, or imaging capacity formed part of the intervention.

Outcome measures

The two primary outcomes were the proportion of inpatient echocardiography requests considered appropriate according to the predefined BSE-based decision rule and the proportion of echocardiograms completed within 24 hours of the recorded request timestamp.

Completion time was defined as the interval between the recorded date and time of the echocardiography request and the date and time of echocardiographic image acquisition/completion. Report authorisation time was not used as the endpoint.

Secondary outcomes included the distribution of requests across the four retrospective BSE urgency categories, the proportion of urgent requests completed within 24 hours, the proportion of emergency requests completed within approximately 60 minutes, and the median time to echocardiogram completion with interquartile range.

Statistical analysis

Categorical variables were reported as numbers and percentages. Absolute differences between baseline and reaudit proportions were reported as percentage-point changes. For the two primary outcomes, 95% confidence intervals for the difference in proportions were calculated using the Newcombe-Wilson method. Fisher's exact test was used to compare proportions. For the retrospectively classified urgent subgroup, the denominator was the number of requests classified as urgent in each audit period. Emergency requests were reported descriptively because of the very small number of observations. The median completion time and interquartile range were calculated for requests with usable exact completion timestamps. Given the uncontrolled before-and-after design and only two measurement points, statistical testing was used to quantify uncertainty around the observed differences rather than to establish causality.

## Results

Sample

A total of 212 requests were screened across the two sampling periods to obtain 200 eligible requests for analysis. During the baseline period, 107 requests were screened, and seven were excluded because the patients had been discharged before audit assessment, leaving 100 requests for analysis. During the reaudit period, 105 requests were screened, and five were excluded for the same reason, leaving 100 requests for analysis.

Primary outcomes

The proportion of requests considered appropriate increased from 82/100 (82%) at baseline to 90/100 (90%) at reaudit, an absolute increase of 8 percentage points. The 95% confidence interval for the difference was −1.7 to +17.8 percentage points, with Fisher's exact p=0.153. The predefined target of 90% was therefore reached by the point estimate, although the observed difference was not statistically significant. Completion within 24 hours increased from 77/100 (77%) at baseline to 79/100 (79%) at reaudit, an absolute increase of 2 percentage points. The 95% confidence interval for the difference was −9.5 to +13.4 percentage points, with Fisher's exact p=0.865. The predefined target of 85% completion within 24 hours was therefore not achieved (Table 1).

**Table 1:** Primary outcomes at baseline and reaudit

Outcome	Baseline, n (%)	Reaudit, n (%)	Absolute change	95% CI for difference	Fisher's exact p
Appropriate request	82 (82%)	90 (90%)	+8 pp	−1.7 to +17.8 pp	0.153
Inappropriate request	18 (18%)	10 (10%)	−8 pp	-	-
Completed within 24 hours	77 (77%)	79 (79%)	+2 pp	−9.5 to +13.4 pp	0.865

Time to echocardiogram completion

Completion time distributions were mutually exclusive and accounted for all 100 requests in each audit period. At baseline, 77/100 (77%) were completed within 24 hours, 10/100 (10%) within 24 to <48 hours, 4/100 (4%) within 48 to <96 hours, and none within ≥96 hours. Nine requests (9%) were not completed, were rejected, or did not have a usable completion timestamp. At reaudit, 79/100 (79%) were completed within 24 hours, 15/100 (15%) within 24 to <48 hours, 6/100 (6%) within 48 to <96 hours, and none within ≥96 hours. No requests were in the not completed/rejected/no usable timestamp category. The median time from request to echocardiographic completion was 14.8 hours (IQR 5.6-21.4) at baseline and 17.5 hours (IQR 3.3-23.4) at reaudit. The baseline median excluded one request for which the completion date was recorded without an exact completion time; this request remained included in the categorical timing analysis (Table 2).

**Table 2:** Time from echocardiography request to completion

Time from request to completion	Baseline, n (%)	Reaudit, n (%)
<24 hours	77 (77%)	79 (79%)
24 to <48 hours	10 (10%)	15 (15%)
48 to <96 hours	4 (4%)	6 (6%)
≥96 hours	0 (0%)	0 (0%)
Not completed/rejected/no usable timestamp	9 (9%)	0 (0%)
Median time, hours (IQR)	14.8 (5.6-21.4)	17.5 (3.3-23.4)

BSE urgency classification

Retrospective adjudication using the BSE framework classified 1/100 (1%) baseline requests as emergency, 71/100 (71%) as urgent, 26/100 (26%) as indicated but non-urgent, and 2/100 (2%) as not indicated. At reaudit, 2/100 (2%) requests were classified as emergency, 77/100 (77%) as urgent, 20/100 (20%) as indicated but non-urgent, and 1/100 (1%) as not indicated. Among retrospectively classified urgent requests, 55/71 (77.5%) were completed within 24 hours at baseline compared with 59/77 (76.6%) at reaudit. The absolute change was −0.9 percentage points (95% CI approximately −14.2 to +12.8 percentage points; Fisher's exact p=1.00).

The emergency subgroup comprised one baseline request and two reaudit requests. The baseline emergency request was completed in approximately 60 minutes. The two reaudit emergency requests were completed after approximately 18.8 hours and 2.3 hours, respectively. Because of the very small number of emergency requests, these findings were reported descriptively without inferential analysis (Table 3).

**Table 3:** Retrospective British Society of Echocardiography urgency classification and category-specific timing

BSE category	Baseline, n (%)	Reaudit, n (%)
Emergency	1 (1%)	2 (2%)
Urgent	71 (71%)	77 (77%)
Indicated but non-urgent	26 (26%)	20 (20%)
Not indicated	2 (2%)	1 (1%)
Urgent completed within 24 hours	55/71 (77.5%)	59/77 (76.6%)
Emergency completed within approximately 60 minutes	1/1 (100%)	0/2 (0%)

Contextual observations

Several operational observations arose during the quality improvement process but were not collected using a formal qualitative methodology and were not quantified as study outcomes.

Requests containing limited written clinical information were sometimes supplemented by verbal communication between referring clinicians and cardiac physiologists (echocardiographers). Repeat echocardiography requests in patients with established cardiac disease also sometimes required consideration of whether the anticipated result would alter immediate management.

High heart rates may affect some echocardiographic measurements and their interpretation, and clinical stabilisation or rate control may therefore be relevant to the timing or quality of imaging in individual patients. These observations were not systematically measured and should be regarded as contextual rather than formal outcome data.

## Discussion

This quality improvement project examined whether a guideline-based educational intervention was followed by changes in inpatient echocardiography request appropriateness and completion timing. The proportion of appropriate requests increased from 82% to 90%, while overall completion within 24 hours changed from 77% to 79%. Because this was an uncontrolled before-and-after study with two measurement points, the observed changes cannot be attributed causally to the intervention.

The increase in request appropriateness is consistent with previous literature demonstrating that educational and decision-support interventions can improve appropriate echocardiography utilisation. Bhatia et al. reported that an educational intervention based on appropriate use criteria reduced inappropriate outpatient transthoracic echocardiogram (TTE) and increased appropriate TTE ordering [3]. Levitt et al. similarly reported improvement in appropriate stress echocardiography following an educational and decision-support strategy [4]. Tao et al. subsequently identified nine studies involving 22,070 patients in a systematic review and meta-analysis and found that quality improvement interventions were associated with a reduction in rarely appropriate echocardiography; education and decision-support interventions were both associated with reduced rarely appropriate testing [5]. The 8-percentage-point increase observed in the present project is directionally consistent with this literature, although direct comparison is limited by differences in populations, definitions, interventions, and study designs.

The findings also support the relevance of improving echocardiography referral information. Kotzé and Parrish identified omissions and inconsistencies in echocardiography referral forms and highlighted the importance of coherent clinical communication [1]. In the present project, the intervention specifically emphasised clinical indication, relevant history and examination findings, and appropriate urgency classification.

The findings regarding timeliness were more limited. Overall completion within 24 hours changed by only 2 percentage points, and the confidence interval included both a clinically relevant decrease and increase. The predefined 85% target was not achieved. The median completion time was also numerically higher at reaudit than baseline. These findings do not support describing overall timeliness as having improved.

Importantly, the BSE 24-hour standard applies principally to urgent studies rather than to all inpatient echocardiograms [2]. Retrospective classification showed that 71% of baseline requests and 77% of reaudit requests were urgent. Completion within 24 hours among these urgent requests remained broadly unchanged, at 77.5% and 76.6%, respectively.

The project therefore does not demonstrate that improved request appropriateness resulted in improved echocardiography timeliness. Completion time is influenced by factors beyond request quality, including clinical urgency, workload, staffing, competing demands, and operational capacity. These service-level factors were not formally measured.

The theory of change proposed that education would improve clinician knowledge, which would improve request quality and potentially facilitate clearer triage. However, clinician attendance, request-return rates, clarification time, weekly request volume, echocardiography staffing, and physiologist workload were not measured. Consequently, the proposed pathway cannot be directly evaluated.

Limitations

This project was a single-centre uncontrolled before-and-after study with only two measurement points. Common-cause variation, secular or seasonal trends, staffing changes, regression to the mean, and other contemporaneous influences cannot be excluded. The sample size was limited to 100 requests in each period. The 8-percentage-point change in appropriateness was not statistically significant, and the confidence interval was wide, indicating limited statistical power. The sampling approach was pragmatic rather than random. Differences in case mix between the two periods may therefore have introduced selection bias. Seven requests were excluded during baseline sampling and five during reaudit because the patients had been discharged before audit assessment. A sensitivity analysis including these requests was not performed.

Appropriateness was assessed by a single cardiology consultant. Although the assessor was blinded to the audit period, there was no second assessor; therefore, there was no inter-rater reliability assessment. The four-category BSE urgency classification was retrospectively adjudicated from written request information rather than obtained from contemporaneous echocardiography-service triage records.

Intervention reach was not formally measured. Although 20 teaching sessions were delivered, attendance was not recorded, and the number and precise locations of posters were not documented. No process or balancing measures were collected. Weekly request volume, physiologist sessions, waiting-list size, request-return rates, staffing changes, and service capacity were not measured. The reaudit was conducted shortly after the intervention, with no longer-term follow-up. Sustainability therefore remains uncertain.

## Conclusions

Targeted clinician education and dissemination of BSE guidance were followed by an increase in the proportion of inpatient echocardiography requests judged appropriate. Overall completion within 24 hours changed only modestly, and compliance among retrospectively classified urgent requests remained broadly unchanged. These findings support continued use of guideline-based education and iterative audit to improve referral quality. Future improvement cycles should incorporate process and balancing measures, evaluate service-level contributors to timeliness, assess intervention reach, and examine whether changes in request appropriateness are sustained over time.
